# Supplementary material for: A set of multi-entry identification keys to African frugivorous flies (Diptera, Tephritidae)
Source: Zookeys. 2014 Jul 24;(428):97–108. doi: 10.3897/zookeys.428.7366 (PMC4143993; doi:10.3897/zookeys.428.7366)
Supplement: Supplementary material 6 — Key to Ceratitis [file zookeys-428-097-s006.zip › SF6_ZooKeys_key to Ceratitis/key/SF6_ZooKeys_key to Ceratitis/Media/Html/Ceratitis guttiformis .htm]

Ceratitis (Ceratalaspis) guttiformis Munro


***Ceratitis*** **(*Ceratalaspis*)
*guttiformis* Munro**

*Ceratitis* (*Pardalaspis*)
*guttiformis* Munro, 1935: 309.

Body
length: 5.80 (5.75-5.85) mm; wing length: 5.03 (4.65-5.40) mm.

Female

Head.
Antenna yellow-orange. Third antennal segment three times as long as second segment.
Arista with longer hairs but not distinctly plumose. Frons with short scattered
hairs which are distinctly darker than frons (in paratype only slightly darker
however); concave to flat, in lateral view not distinctly projecting forwards
at antennal implant. Chaetotaxy normal for subgenus.

Thorax.
Postpronotum concolorous with ground colour of mesonotum, with black spot.
Ground colour of mesonotum pale with orange tinge. Chaetotaxy normal for
subgenus. Scapular setae black. One anepisternal bristle. Scutellum white
basally, otherwise yellow with three black separate markings apically; basally
with 2 dark spots (poorly developed in holotype). Subscutellum pale with three
dark separate spots.

Legs
yellow; setation typical for subgenus, mainly pale especially on femora.
Posterior and posterodorsal rows on fore femur pale.

Wing
bands with markings extensively yellow. Banding, marginal band continuous;
cubital band free or very weakly connected with discal band; medial band
absent; crossvein r-m at or before middle of discal cell. Crossvein dm-cu
posteriorly more outwards than anteriorly.

Abdomen.
Yellow-orange colour. Setation and banding typical for subgenus. Oviscape
almost as long as abdominal terga 3-6 combined.�

Male

Unknown.

(Description
after De Meyer, 1998)
